# Childhood exposure to domestic violence: can global estimates on the scale of exposure be obtained using existing measures?

**DOI:** 10.3389/fpubh.2024.1181837

**Published:** 2024-05-22

**Authors:** Rebecca Jane Harris, Andrew Amos Channon, Sara Afshar Morgan

**Affiliations:** ^1^Centre for Global Health and Policy, Department of Social Statistics and Demography, University of Southampton, Southampton, United Kingdom; ^2^Centre for Population Health Sciences, Faculty of Medicine, University of Southampton, Southampton, United Kingdom

**Keywords:** children, childhood, domestic violence and abuse, intimate partner violence, exposure, measurement scales, cross-cultural comparisons, LMIC

## Abstract

**Purpose:**

Childhood exposure to domestic violence and abuse (DVA) can lead to major short- and long-term effects on the victim. Despite this, there is no accepted measure for children’s experiences, with most existing measures being validated only in high income countries and not in low- and middle- income countries. As a result, international statistics are not comparable. This paper seeks to critically appraise existing measures and discuss whether any are fit-for-purpose on a global scale.

**Method:**

The COSMIN PROMs approach was followed to critically appraise and compare the appropriateness of measures. A comprehensive literature search was undertaken in seven journal databases for measures mentioned in formally peer-reviewed articles exploring childhood exposure to DVA.

**Results:**

A literature search resulted in the identification of 10 measures and, following criteria to only keep original measures and remove modifications, four measures which have been validated cross-culturally are discussed in detail in line with the COSMIN PROMs criterion: The Child Exposure to Domestic Violence Scale, Children’s Perception of Interparental Conflict Scale, Juvenile Victimization Questionnaire and The Violence Exposure Scale for Children. Strengths and limitations of each are discussed, along with any validations undertaken not in the country of origin.

**Conclusion:**

Despite childhood exposure to DVA being an urgent research priority worldwide, the current measures to explore the extent of the issue are not validated cross-culturally, leading to concerns about comparisons across different population groups. The development and implementation of interventions to reduce the levels and effects of exposure relies heavily on cross-cultural comparisons, which may indicate different strategies are needed in different contexts. The lack of these validated comparisons is constraining advances, and the paper advocates for further efforts to be made in this regard.

## Introduction

Childhood exposure to domestic violence and abuse (DVA) is a concept that is increasingly understood to have major short- and long-term effects on the victim (1). Globally, there has been an observed increase in DVA around the world following the responses to COVID-19 (2), with studies indicating an associated increase in the numbers of children exposed to DVA within a home environment (3). However, identifying those children who are exposed to DVA is complicated by a lack of agreement on appropriate ways to measure exposure to DVA, with a range of different tools and measures available. Despite being a global epidemic (4), many of the available measures for childhood exposure (also referred to as ‘experience’ in some literature) have only been tested or used in higher-income settings, leading to concerns around their applicability to different contexts and countries (5).

Existing global estimates on the scale of childhood exposure to DVA are potentially problematic, leading to inconsistent measurements over time. As a result, this form of violence against children does not receive the necessary attention among policymakers. Without a credible baseline indicator, it is difficult to argue for change; it means that international organizations, including multilateral organizations and regional donors, are unable to properly assess the need for intervention (6, 7). An absence of an appropriate measure raises challenges in estimating the global scale of the issue, and in observing and tracking the social impact of policies and interventions related to child protection and exposure to DVA. This means that international statistics are not comparable, thereby limiting its visibility within international bodies, and the ability to identify countries underperforming in the reduction of childhood exposure to DVA.

Based on a comprehensive literature search, the most recent review of measures exploring childhood exposure to DVA is by Latzman et al. (5), although this review did not explicitly focus on the validation of available measures, nor explore their applicability globally. Additionally, the study of childhood exposure to DVA has advanced significantly in the past seven years, with a number of countries now recognizing children within DVA legislation [e.g., (8, 9)].

Despite the majority of research on childhood exposure to DVA being conducted in high income countries (HICs), several factors mean that children could be more likely to be exposed to DVA in low- and middle-income countries (LMICs) (4). The socioeconomic status of a country has been linked to higher rates of DVA, while it is also known to be related to poverty and economic inequality. Current research suggests that the prevalence of both DVA and violence against children in LMICs is higher than in HICs (10, 11), although the evidence base is sparse. However, there is evidence to suggest that children in LMICs may spend more time at home compared to children in high-income countries (12), meaning that if violence were to occur in their home, children in LMICs are at an increased risk of exposure. Therefore, it is essential to ensure that measures for childhood exposure to DVA are applicable on a global context, particularly within LMICs.

The aim of this review was to examine the current measures that have been developed to measure childhood exposure to DVA, to critically appraise these in order to understand if they can be used to obtain global estimates of the numbers and scale of those affected, with a particular focus on LMICs.

### Childhood exposure to domestic violence and abuse

Domestic violence and abuse is defined as an incident or pattern of incidents of controlling, coercive, threatening, degrading and violent behavior (13). In the majority of cases, this is inflicted by a partner or ex-partner, but it can also be by a family member or carer (14). This violence can be physical, sexual, emotional, or psychological, as well as involving violence such as economic coercion (15, 16). Whilst DVA commonly refers to violence within the home between caregivers, in some countries and studies, child or elder abuse, or abuse by any member of a household is also considered DVA (17). For the purpose of this paper, the term domestic violence and abuse will be used, as this is more inclusive than other terminology which is often used interchangeably (e.g., ‘intimate partner violence’, or ‘marital violence’), and encompasses all forms of violence and abuse that occurs between adults in a home which a child could be exposed to.

The concept of childhood exposure to DVA, and the psychological impact that this has on an individual, is a relatively new phenomenon when considered within the wider research area of DVA (4). Earlier researchers often describe the child or adolescent as an ‘observer’ or ‘witness’, but later research instead takes a more child-centric approach, saying that they are ‘exposed to’, ‘affected by’ or ‘experience’ the violence, as this is more inclusive, and does not assume that the individual actually observed the violence whilst it was happening (18, 19). For clarity, in this study the terms ‘exposed/exposure’ will be used, covering all the approaches noted above.

Whilst there is limited information on exposure in LMICs, evidence from HICs indicates that children who are exposed to DVA are at an increased risk of psychiatric disorders, substance misuse, and are more likely to commit violent crimes in adulthood (20, 21). There is also a wealth of evidence to support the ‘intergenerational transmission of violence’ (22), which suggests that children exposed to DVA are more likely to become either perpetrators or victims of DVA later in life when compared to those who were not exposed to DVA in childhood (4, 23, 24). In some countries, childhood exposure to DVA is considered a form of child maltreatment or neglect (9, 25), as the child is living in a psychologically abusive, physiologically arousing, emotionally distressing, and often trauma inducing environment (26, 27).

### Measuring childhood exposure to DVA

The most recent global estimates available found that close to 300 million children (defined as those aged under 18 or the relevant age of majority in each country) are exposed to DVA worldwide (4). However, these figures were obtained through reviewing existing studies that measure violence in the home in various countries and did not use a standardized tool. This, therefore, may have led to under or over-reporting, and the estimates may not be accurate. These 2006 UNICEF estimates also did not provide estimates for the regions of Northern Africa, or South-Eastern Asia, accounting for the wide-ranging figure. Higher estimates for the number of children who are exposed to DVA within these regions could be due to a number of factors, such as cross-cultural variations in living conditions, a lower socioeconomic level (where physical violence is often found to be higher), and due to there being a higher level of violence in society, which is often seen reflected at a household level (28).

Additionally, although no recent global estimates have been found, any more recent estimates of childhood exposure to DVA would likely be higher due to the increase of DVA reported around the globe due to the ‘shadow pandemic’ (of DVA) caused by the imposed lockdowns due to COVID-19 (2, 29). A 2020 report by the United Nations Population Fund (UNFPA) stated that the COVID-19 pandemic may result in an additional 31 million cases of gender-based violence worldwide if lockdowns were to continue for 6 months, and for every 3 months that the lockdown continued, an additional 15 million additional cases were expected (30). Increased stress, disruption of social and protective networks, closure of schools, restrictions on movement, economic insecurity, and decreased access to services can all exacerbate the risk of violence for women and increase a child’s likelihood of being exposed to DVA (29), a fact reflected in studies since the start of the pandemic (3). Childhood exposure to DVA and obtaining global estimates is, therefore, an urgent research priority.

Despite childhood exposure to DVA being an important issue, there are a number of methodological limitations which hinder research into this area. Childhood exposure to DVA is not a dichotomous concept of whether or not a child has witnessed or overheard violence, and within the literature, there are varying thresholds researchers use to consider a victim ‘exposed’ to DVA (18), with a number of studies failing to provide descriptions of the nature of the victim’s exposure within their methodologies (31). Factors such as the frequency, duration, severity, and level of exposure (i.e., saw the violent act, heard about it from another room, or learned about it later), all have the potential to influence classification, along with the timeframe in which they are exposed to the DVA (1).

### Current review

While some theoretical and critical reviews of both childhood exposure to DVA and its measurement strategies exist [e.g., (5, 32)], no review to date has focused on the suitability or applicability of these measures in a global context, particularly within LMICs.

The first aim of this paper was to provide an overview and critique of existing measures for childhood exposure to DVA, to discuss the usage of these existing measures over time and their validations cross-culturally. The second aim was to discuss key limitations in the field of measuring childhood exposure to DVA and make suggestions for future improvement of research in the area within LMICs, focusing on how these measures can support the development of appropriate interventions within the health system, if validated.

## Methods

There are currently no tools for critically appraising and comparing the appropriateness of measures in different contexts, particularly in respect to measuring childhood exposure to DVA. Therefore, we used the COSMIN (COnsensus-based Standards for the selection of health Measurement INstruments) approach and criterion for Patient-Reported Outcome Measures (PROMs) (33).

COSMIN PROMs provides a framework for evaluating the methodological quality of outcome measures, through evaluating their measurement properties. It is a systematic approach to assessing the quality of the measurement properties of these instruments, such as reliability, validity, and responsiveness. When compared to other potential tools, the COSMIN PROMs criteria includes cross-cultural validity as a criterion, making it an appropriate tool for this paper.

### Search strategy

The following electronic databases were searched: Directory of Open Access Journals, JSTOR, PsycINFO, PubMed, Science Direct, Scopus, and Web of Science. Abstracts were searched for terms related to age (e.g., child*, youth, teen*, juvenile, adoles*), exposure (e.g., expos*, witness*, observ*), DVA (e.g., IPV, domestic violence, domestic abuse), and those related to measurement (e.g., tool, survey, scale, index, questionnaire, assess*, measur*).

### Inclusion criteria

Measures mentioned in formally peer-reviewed articles that have been published in indexed journals were included. Gray literature such as blogs, unpublished dissertations, and unofficial reports were excluded, as despite some measures being mentioned in these, there is limited information about them. Measures exploring childhood exposure to DVA in any capacity were considered, such as self-reported and caregiver reported measures, and included if they measured exposure to any additional forms of violence, e.g., exposure to community violence. Modifications of established measures were examined individually by the research team, and included in the results *only* if the modifications that had been made were related to the aims of this study. Revisions of original measures by the same authors were included, where appropriate. This was to ensure consistency across the approach to this study, and to ensure a fair comparison of published measures to be considered.

From these resulting papers, the researchers examined abstracts and removed any that did not adhere to the inclusion criteria, and noted the measures used within the remaining papers. Measures that have been applied cross-culturally were examined closely to answer the aim of this research.

## Results

The literature search led to the identification of 10 measures that focus on child exposure to DVA, summarized in Table 1 (further detail on each of these is available in Appendix A). The majority of these measures have not been used or applied in countries other than that of their original high-income setting; only four measures that have been applied cross-culturally were identified. These were the Child Exposure to Domestic Violence Scale (CEDV), Children’s Perception of Interparental Conflict Scale (CPIC), Juvenile Victimization Questionnaire (JVQ), and the Violence Exposure Scale for Children (VESC).

**Table 1:** A summary of identified measures.

Measure name	Year of publication	Country of publication/origin	Applied outside of country of origin?	Applied in LMIC?
Caregiver-report Modified version of the Severity of Violence Against Women Scales (34)	2011 Adaptation of Marshall (35)	United States	No	No
Child Exposure to Domestic Violence Scale (36)	2008	United States	Yes	Yes
Childhood Experiences of Violence Questionnaire (37)	2008	Canada	No	No
Children’s Perception of Interparental Conflict Scale (38)	1992	United States	Yes	Yes
Computer Assisted Child Maltreatment Inventory (39)	2010	United States	No	No
Family Aggression Screening Tool (40)	2016	United Kingdom	No	No
Juvenile Victimization Questionnaire (41)	2005	United States	Yes	Yes
Things I Have Seen and Heard (42)	1993	United States	No	No
Timeline Followback Interview – Children’s Exposure to Partner Violence (43)	2009	United States	No	No
Violence Exposure Scale for Children (44)	1995	United States	Yes	No

The four measures that have been applied cross-culturally are discussed in further detail below and illustrated in Table 2, which also contains some key criteria from COSMIN PROMs (33), including an evaluation of content validity, internal consistency, cross-cultural validity and construct validity, where this information was available. A full summary of indicators with relevant studies can be found within Table 2, and are summarized below:

- Cross-cultural validity was assessed for all measures, and all four have been applied within countries other than their origin.
- Information on content validity could not be found for the JVQ, but the CEDV, CPIC and VESC were all found to have content validity when compared with other measures.
- Internal consistency was assessed for all four measures, with the CEDV and JVQ reporting good internal consistency, and the CPIC and VESC reporting adequate to good internal consistency.
- Information on construct validity could not be found for the CPIC, and for the JVQ, construct validity was found but not for exposure to DVA. For the CEDV and VESC, convergent, concurrent and discriminant validity was found.
- The majority of studies exploring reliability used test–retest methods, and found correlations for all scales, with the highest correlation being found for the CEDV.

**Table 2:** Summary of childhood exposure to DVA measures applied cross-culturally by COSMIN PROMs indicators.

Measure	Content validity	Internal consistency	Cross-cultural validity	Reliability	Construct validity
Child Exposure to Domestic Violence Scale (CEDV) (46)	A panel of nine experts who work with children exposed to parental IPV were asked to review the measure during the creation of the CEDV. The experts were asked to examine each item and suggest whether to keep the item ‘as is’, delete the item from the measure, or revise the question. This increases content validity (47).	Good levels of internal consistency have been reported, ranging from α = 0.79 (48) to α = 0.97 (49).	Brazil (50) India (51) Iran (52) Iraqi-Kurdistan (53) Pakistan (54) South Africa (55) Spain (56) Sweden (48, 57, 58)	Test–retest reliability was assessed by calculating Pearson’s correlation coefficients and paired t-tests between Week 1 and Week 2 (46). Pearson’s correlation coefficients for each subscale ranged from 0.57 to 0.70 (*p* < 0.001), indicating relatively strong and statistically significant correlations between the two administrations. Non-significant differences on t-tests between administrations also showed that Week 1 and Week 2 test scores for the level of violence in the home, home exposure, community exposure, risk factors, and other victimization were very similar and stable over the two administrations. The exception was the “level of involvement” subscale, which produced highly correlated answers but showed significantly different test–retest scores in the paired t-test (*t* = −2.154, *p* < 0.05). Test–retest reliability in other studies has found Pearson’s correlation coefficients between the two administrations of *r* = 0.58–0.89 and for the total scale, *r* = 0.86 (53). None of the tests demonstrated significant differences between time one and time two.	Convergent validity was assessed by asking each child to complete the TISH questionnaire (45) along with the CEDV (46). Correlations were found between violence in the home and violence in the community subscales (46). Concurrent validity was explored by examining the correlation coefficients of scores on the CEDV and a questionnaire on exposure to physical aggression (52). Results indicated that subscales of exposure to parental IPV on each measure were positively correlated (52). Grip et al. (48) examined the relationship between the CEDV, and the Strengths and Difficulties Questionnaire (SDQ) and Trauma Symptoms Checklist for Children (TSCC). The TSCC included a post-traumatic stress scale, and the mean of the t-scores on the additional scales were used to operationalize psychological problems in general. The CEDV was not significantly related to any of the scales, indicating discriminant validity (49).
Children’s Perception of Interparental Conflict Scale (CPIC) (38)	The CPIC was developed against two established measures of parent-reported interparental conflict, the Conflict Tactics Scale (59) and the O’Leary Porter Scale (60). The CPIC has been found to be significantly related to these measures, with Pearson’s correlation coefficients *r* = 0.30 and *r* = 0.39, respectively (38).Whilst these are moderate correlations, Grych et al. (1992) justified it as such measures do not assess child adjustment and are completed by parents and not the child (38).	Three factor analytically derived subscales (Conflict Properties, Threat, Self-Blame) were assessed in the original paper (38). Alpha coefficients were computed, and each subscale demonstrated good internal consistency across samples, with all values greater than α = 0.70. Several studies have used the CPIC and, in most cases, have shown adequate internal consistency (61–63).	China (64–66) Pakistan (67) Portugal (68) Spain (69)	To assess test–retest reliability, 44 children were measured for the three factor analytically derived subscales two weeks after the initial administration of the test (38). Conflict properties *r* = 0.70, Threat *r* = 0.68, and Self-Blame *r* = 0.76. Inter-scale correlations were also assessed, with the Conflict Properties scale, and Self-Blame scale items being highly correlated, and the Threat scale being moderately correlated.	No information found.
Juvenile Victimization Questionnaire (JVQ) (41)	No information found.	Internal consistency not measured specifically for items related to exposure to DVA, but for the whole tool, internal consistency was good, α = 0.80 (41).	Australia (70) China (71) Israel (72) Pakistan (73) Portugal (74) South Africa (75) Spain (76) UK (77)	200 of the respondents were re-contacted and re-administered the JVQ within 3–4 weeks of its original administration. This re-test sample included both 100 youth self-respondents and 100 caregiver proxy respondents. The instrument showed adequate test–retest reliability (41). The mean κ was 0.59 with a range from 0.22 to 1.00. For the self-reporting youth, the mean κ was 0.63, with range of 0.22–1.00 and for the caregiver proxies the mean κ was 0.50, range −0.03 to 1.00 (41).	Evidence of construct validity, but not from studies measuring exposure to DVA.
Violence Exposure Scale for Children (VESC) (44)	The researchers describe the study as “face valid” and as having content validity as it was developed on the TISH questionnaire (45) and asks about similar areas of violence exposure to other measures.	Internal consistency: α = 0.72 to 0.86 (78).	Israel (79) The measure is also available in Spanish [according to (80)], although no studies using this measure could be found.	There are no interrater statistics for interviews. A study comparing parent’s and children’s reports of the child’s exposure to violence found a significant relationship (81).	Convergent and concurrent validity was found in non-clinical and diverse samples, and discriminant validity found for diverse samples (78).

Despite not meeting the criterion for further discussion (due to not being applied cross-culturally), a measure the researchers thought was important to acknowledge from Table 1 is the Things I Have Seen and Heard Scale (TISH) (45). This measure was designed to measure the level of direct and indirect exposure to violence that children experience at home, as well as in the wider community. The TISH has demonstrated relatively strong internal consistency, with Cronbach’s Alpha between α = 0.74 and α = 0.76. High reliability has also been established through strong test–retest (*r* = 0.67) and inter-rater reliability results (*r* = 0.81) (42). The TISH is an important measure within this area of research, with other researchers either using it as a foundation for the development of their own measure, or as a tool to establish validity of their own measure (as discussed below and in Table 2).

### Child exposure to domestic violence scale

The CEDV scale, developed by Edleson et al., is a 42-item self-administered measure (46). The measure was developed using several existing surveys and interview guides investigating childhood exposure to DVA, based on key content areas identified in a 2007 review by Edleson et al. (36). Nine international experts working with children who have been exposed to DVA were then invited to review each item and suggest whether it should be kept without changes, deleted from the measure, or if the question should be revised. They were also offered the opportunity to advise any additional items or content that should be included in the measure. The CEDV asks children aged 10–16 to answer questions using a Likert-type scale, with the choices being “never,” “sometimes,” and “a lot.” If the child chose one of the latter two options, indicating that the violent event occurred at home, they were directed to another set of questions which asked how the child was exposed to the DVA, including five choices: “I saw the outcome (e.g., someone was hurt, something was broken, or the police came),” “I heard about it afterwards,” “I heard it while it was happening,” “I saw it from far away while it was happening” and “I saw it and was near while it was happening.” The second section of the CEDV asks a series of 23 questions using the same three-point Likert-type scale, and they were asked about how often they intervened in violent events and about other risk factors present in their lives (e.g., substance use in the home), and the third section collected demographic information including age, gender, race, ethnicity, current living situation, and family composition. The questionnaire was tested amongst 65 children aged 10 to 16 (M = 12.5, SD = 2.1) staying in domestic abuse shelters with their mothers—a potential methodological issue as this sample is not representative of the general population. When compared to the TISH for convergent validity, the correlation between the CEDV and TISH indicated that a statistically significant and positive correlation existed both at the level of home violence exposure (*r* = 0.494, *p* < 0.001) and community violence exposure (*r* = 0.397, *p* < 0.001) (45).

It was hoped that as a measure, the CEDV would be able to address the shortage of empirically validated measures for childhood exposure to domestic violence (1). According to Holden’s taxonomy (18), the CEDV addresses several categories of exposure to DVA, including witnessing, overhearing, observing the initial effects, hearing about it, and intervening.

Ravi and Tonui conducted a systematic review to synthesize and summarize the psychometric properties of the CEDV to assess the reliability and validity of the measure (47). From an initial identification of 264 studies using the measure, 13 studies were used as the final sample in the review after removing duplicates and applying the inclusion criterion. The final studies, when combined, had a total sample size of 2,546 children (Mean age = 12.70, SD = 3.30), that ranged across countries (including a mixture of HICs and LMICs), with the measure being translated and administered into several languages. This includes Sweden (48, 57, 58), India (51), Spain (56), Brazil (50), Iraqi-Kurdistan (53), Pakistan (54), South Africa (55) and Iran (52). Despite being translated into different languages for implementation in different countries and contexts, internal consistency remained when utilizing the CEDV with diverse populations, and the results indicated that the CEDV demonstrated content, convergent, and discriminant validity (47). However, there were some inconsistencies regarding the association of childhood exposure to DVA with internalizing problems such as mental health concerns or depression symptoms (49, 82). Limitations with factor and concurrent validity were also identified, and Ravi and Tonui suggested that future research should employ exploratory factor analysis to examine the factor structure of the measure when it is used with various populations, and that future studies should use larger sample sizes to understand more about key correlates and latent variables (47).

### Children’s perception of interparental conflict scale

The CPIC, developed by Grych et al. (38), is a 48-item self-report measure designed to assess multiple dimensions of marital conflict that might lead to child adjustment problems, and to obtain children’s perspectives on the degree of the conflict to which they are exposed, including questions about witnessing, overhearing, and interfering in the conflict. The developers’ motivations for developing this measure were based on the fact that “parent-report measures often underestimate children’s exposure to conflict” (38). Children are given a statement, for example, “my parents have pushed or shoved each other during an argument,” and they are asked whether this statement is “false,” “sort of true,” or “true.”

The CPIC was designed for children aged 9–17 years old (38), but further studies have found the measure to also be appropriate for young adults aged 18–25 years (63) and has also been implemented in countries other than its origin (United States), including China (64–66), Pakistan (67), Portugal (68), and Spain (69). The measure and scoring manual are free and easily accessible online, which promotes wider use, especially in resource-restricted countries or locations.

The subscales of the CPIC assess frequency, intensity, resolution, content, perceived threat, coping efficacy, self-blame, triangulation and stability—a comprehensive and detailed approach to understanding the DVA that a child is exposed to. Three broad-factor scales were developed using exploratory factor analysis and confirmatory factor analysis. The first of these is Conflict Properties, which reflects how often conflicts occur and the level of hostility and resolution and comprises of the frequency, intensity and resolution subscales. The second is Threat, which indicates the degree to which children feel threatened and able to cope when marital conflict occurs, which comprises of the threat and coping efficacy subscales. The final broad-factor scale is Self-Blame, which assesses the frequency of child-related conflict and the degree to which children blame themselves for marital conflict, and comprises of the content and self-blame subscales. The stability and triangulation subscales can be used as independent subscales.

Alpha coefficients were computed, and each subscale demonstrated good internal consistency across samples, with all values greater than α = 0.70. Several studies have used the CPIC and, in most cases, have shown adequate internal consistency (61–63). Test–retest reliability was assessed in the original study, with 44 children being assessed for the three factor analytically derived subscales two weeks after the initial administration of the test (38). Conflict properties *r* = 0.70, Threat *r* = 0.68, and Self-Blame *r* = 0.76, indicating moderate to high correlations. Inter-scale correlations were also assessed, with the Conflict Properties scale and Self-Blame scale items being highly correlated, and the Threat scale being moderately correlated. This demonstrates that the CPIC is a reliable measure in assessing the child’s perception of parental conflict, an important outcome that is often not assessed by other measures exploring childhood exposure to DVA. However, Pote et al. found insufficient evidence that the CPIC is a valid measure when investigating changes in childhood exposure to DVA in short interventions (less than 3 months), which is a potential limitation of the measure (83).

### Juvenile victimization questionnaire

The JVQ, developed by Finkelhor et al. (41) is a 37-item questionnaire exploring various forms of juvenile victimization, including witnessing violence and indirect victimization. The questionnaire aims to measure multiple forms of juvenile victimization to obtain a better estimate of the total rate of victimization, enhance the correspondence of juvenile victimization measurement with important social constructs such as crime and child protection categories, and to provide a means of studying the overlap among forms of juvenile victimization.

The JVQ can be used in an interview format for children 9–17 years old, and self-administered for juveniles aged 12 and over. There is also a caregiver version, where a caregiver can be interviewed by proxy for a child, for example, if a child is under the age of eight. Additionally, it can be adapted for retrospective reporting of childhood events for completion by adult respondents, making the questionnaire versatile when exploring childhood exposure to DVA (41). The authors argue that the JVQ offers enhanced opportunities to obtain accurate epidemiological data across childhood, because it can be administered in self-report form from the age of eight, which is younger than previous measure allowed (84).

The JVQ consists of modules, including Conventional Crime, Child Maltreatment, Peer and Sibling victimization, Sexual Victimization, Witnessing and Indirect Victimization. Follow-up questions are asked for those questions in which a child or caregiver reports that a victimization occurred. Follow-up questions include the number of times a child has been victimized, who victimized the child, whether the child was hurt, and questions specific to the victimization reported (41).

The questionnaire has good internal consistency (α = 0.80) and has been administered in China (71), Israel (72), Pakistan (73), Portugal (74), South Africa (75), Spain (76), and the United Kingdom (77), and has recently been adapted for use in a national study in Australia (70). The measure also demonstrates adequate test–retest reliability, with 200 of the respondents being re-contacted and re-administered the JVQ within 3–4 weeks of its original administration. This re-test sample included both 100 youth self-respondents and 100 caregiver proxy respondents (41). The mean Cohen’s kappa coefficient was κ = 0.59, with a range from κ = 0.22 to κ = 1.00. For the self-reporting youth, this was κ = 0.63, with a range of κ = 0.22 to κ = 1.00, and for the caregiver proxies, the mean was κ = 0.50, range κ = −0.03 to 1.00 (41). There is also evidence of construct validity in studies comparing juvenile victimization with psychological and sociological constructs such as depression and neighborhood crime rates (84).

### Violence exposure scale for children

The VESC, developed by Fox and Leavitt (44), is a 25-item self-report measure of violence exposure that includes drawings to accompany questions, and a Likert-type rating scale. The measure includes questions about minor and severe violence victimization and witnessing violence in the home, school and neighborhood. Children are given a statement, for example, “Chris sees a person beat up another person. How many times have you seen a person beat up another person?” and they are asked if, over the course of their lifetime, this has “never” happened, it has happened “one time,” “a few times,” or “lots of times.”

The VESC was designed for children aged 4–10 years old, with Fox and Leavitt’s initial sample consisting of 40 Caucasian, primarily suburban children (44). The measure has also been administered to 155 African American preschool aged children from low socio-economic status families in Washington DC (81), and following translation to Hebrew, the measure has also been administered to 134 second and fourth-grade students in Israel (79). The measure has also been translated to Spanish (80), although no published studies using this adaptation could be found. An alternate version of the questionnaire is available, with the characters depicted as either male or female, and for the Israeli population, the name of the cartoon character was changed, and several items were altered (79).

Despite this measure being applied in a country other than its origin, the psychometric data available is limited, meaning that it scores low on the COSMIN PROMs criterion (33). The researchers describe the study as “face valid” and as having content validity as it was developed on the TISH (42) and asks about similar areas of violence exposure as other measures. Internal consistency was also reported to be between α = 0.72 to α = 0.86. There are no interrater statistics for interviews. Shahinfar et al. compared parent’s and children’s reports of the child’s exposure to violence and found a significant relationship, but found poor concordance between VESC parent and child reports (81). However, this may be due to differences in interpretation of items or lack of parental knowledge of their child’s exposure. Convergent and concurrent validity was found in non-clinical and diverse samples, and discriminant validity found for diverse samples (78).

## Discussion

The four measures applied cross-culturally were compared using the COSMIN PROMs criterion (33). Information on structural validity, measurement invariance, measurement error, criterion validity, and responsiveness could not be found for any of the measures, so they were not included in the discussion or in Table 2. While this could be considered a potential limitation, it was expected that not all criterions would be used due to COSMIN PROMs being a tool to compare patient-reported outcomes, rather than one to compare measures for childhood exposure to DVA.

When considering the appropriateness of measures for gaining global estimates of childhood exposure to DVA, the researchers considered three main areas: firstly, which measure had been most commonly applied within LMICs, second, which was most acceptable according to the indicators covered in COSMIN PROMs, and finally, which measure covered aspects of exposure to DVA the closest, based on our current understanding. Three of the measures have been applied in LMICs—the CEDV, which has been implemented in Brazil (50), India (51), Iran (52), Iraqi-Kurdistan (53), Pakistan (54), and South Africa (55), the JVQ, which has been applied in China (71), Pakistan (73) and South Africa (75), and the CPIC, which has been applied in China (64–66), and Pakistan (67). Based on this information, it could be assumed that the CEDV is the most appropriate measure for assessing childhood exposure to DVA within LMICs, although it is still sparsely implemented.

However, just because a scale has been applied the most within a certain context does not mean that it is the most appropriate, and so, therefore, the measures need to be examined under the lens of the COSMIN PROMs indicators (Table 2). From this examination, there is heterogeneity in the information relating to the different types of validity, with the CEDV showing cross-cultural, content, internal and construct validity reported. The VESC also reports these measures, but had lower internal consistency. There were gaps in these aspects for the JVQ and CPIC, although these may be available in gray literature or other reports that were not included in this study.

These measures need to be considered in line with the current understanding of childhood exposure to DVA. As previously mentioned, this is a relatively new area of research, and there has already been changes in the way in which childhood exposure to DVA is understood and explored (for example, the change in terminology from ‘witnessed’ to ‘exposed’). Holden’s taxonomy (18) provides a comprehensive overview of how children can be exposed to DVA, and so each of the four measures should be explored to see how aligned they are with these definitions and categories, along with that of the current definition of DVA (13–17). Appendix A provides a summary of the measures, which includes the types of DVA and the type of exposure measured. The VESC has the most constrained definition of exposure to DVA, exploring only physical violence that a child witnesses. The JVQ explores only witnessing and indirect victimization, but measures physical, sexual, and psychological/emotional violence and abuse as exposure types. The CPIC measures the same types of DVA as the JVQ but allows for the exploration of a few more of Holden’s taxonomies – witnessing, overhearing, and intervening. The measure which allows for the most comprehensive definition of both DVA and exposure is the CEDV. The CEDV investigates physical and psychological violence and abuse, and coercive control. It also has questions related to whether a child is an eyewitness, overhears, observes the initial effects, hears about, or intervenes with the violence or abuse.

Based upon the three criteria defined by the researchers, the most comprehensive scale is the CEDV, as this has been applied the most within LMICs, has the highest score using COSMIN PROMs indicators, and allows for exploration of childhood exposure to DVA in a way most closely aligned with the current understanding of the area. This could, in part, be due to the fact that it is the most recent out of the four scales discussed in further detail in this study, and so it was likely developed with a larger body of evidence in mind. However, its application is still sparse, and further research is needed to assess whether it would be applicable and valid across multiple LMICs, and if it could be used globally to gain estimates of childhood exposure to DVA.

Whilst all the measures discussed have strengths, there is no standardized approach to assessing the issue of childhood exposure to DVA, and rather than a consensus being discussed around the best measure for exposure to DVA, modifications to existing measures are often made instead to address the research questions or requirements. These modifications could reflect the changing nature and understanding of DVA within wider society, for example, making questions around perpetrators of violence non-gender specific and recognizing additional forms of DVA, including psychological, emotional, and economic abuse (whereas earlier measures had a primary focus on physical and sexual violence), but it could also be because there is not one accepted measure which is fit for purpose. For larger, country-wide studies, such as the Violence Again Children Surveys (VACS) (85), researchers tend to create their own questionnaires, which contain sections on childhood exposure to DVA, rather than use existing measures. This may be due to space, as most of the above scales have a large number of items, which may make them unfeasible to be used in a broad multi-country survey when trying to gain a global overview of the issue.

The lack of a cross-culturally validated instrument to measure childhood exposure to DVA is likely to be compromising the development of interventions to reduce the burden of exposure or to mitigate the effects. The understanding of the scale of the issue alongside a validated method to assess the success of interventions will support policymakers, and further allow cross-national learning on the effectiveness of interventions that have been implemented. With the increasing number of laws that note childhood exposure to DVA, this is an urgent priority. Without validated baseline data, progress in this area is likely to be limited.

Limitations in measures and how well they are applied, particularly within the LMIC context, have implications on our ability to make accurate estimates of the number of children exposed to DVA. Whilst the increasing interest in exploring childhood exposure to DVA is a positive development, if a standardized measure was available, it would allow for international comparable statistics, ensuring that there is visibility within international bodies to identify countries underperforming in the reduction of childhood exposure to DVA. The most recent global data on child exposure to DVA was published in 2006, when it was estimated that between 133 and 275 million children are exposed to DVA worldwide (4). The estimates indicated a significantly higher number of children are exposed to DVA in LMICs in comparison to HICs, and revealed that there are currently no estimates for the regions of Northern Africa or South-Eastern Asia, accounting for the wide-ranging estimate (4). Accurate statistics also allow for the social impact of policies related to child protection and exposure to DVA to be observed and tracked, and without a credible indicator (baseline), then it is more difficult to argue for change and assess the need for interventions (6). A reliable measure for childhood exposure to DVA could encourage a move toward inclusion of this topic in international goals such as the Sustainable Development Goals, for example, under 5.2: “Eliminate all forms of violence against all women and girls in the public and private spheres, including trafficking and sexual and other types of exploitation” or 16.2: “End abuse, exploitation, trafficking and all forms of violence against children” (86).

A major limitation in this area of research is that when compared to HICs, there is still a lack of studies measuring childhood exposure to DVA in LMICs. It is possible that some data has been collected in LMICs using the measures, but these studies have not led to peer review publication, and so, therefore, they have not been included in this review. Additionally, the use of these measures within the LMIC context has not been validated, and so results from these studies should be generalized with caution. Large-scale studies which measure childhood exposure to DVA (such as the VACS) also opt to use their own questions, as opposed to using measures specifically designed to explore childhood exposure to DVA, raising the question of whether the estimates we have for childhood exposure to DVA are valid and highlighting a need for additional research to be conducted in the field of childhood exposure to DVA. Future research should focus on collating studies measuring childhood exposure to DVA within LMICs to allow for a wider perspective on the measures used and their appropriateness in the LMIC context, along with further development of the measures mentioned (including those not yet applied or validated in LMICs, such as the TISH).

Whilst efforts were made to present a widespread and representative body of literature through database searches, given the increasing body of work on childhood exposure to DVA, it is possible that not all measures for childhood exposure to DVA have been included and discussed, especially if only included in gray literature at the present time. Instead, those most commonly used within existing, published, peer-reviewed literature were included. Another limitation of the present study is that only English-language articles were included in the search process, potentially excluding any articles published in non-English language journals, which may be more likely to contain studies which have applied measures in LMIC settings. However, no references to any articles such as these were found during the search and review for this study.

## Conclusion

Despite it being an urgent research priority, there is no standardized approach or measure for childhood exposure to DVA, a situation which is exacerbated in LMICs. Many of the measures have not been validated in LMICs, leading to gaps in data and practice, and to an understanding of the scale of the issue. For such a sensitive area which already experiences significant underreporting (87), it is paramount that a measure which can be used globally (particularly within LMICs) is agreed upon. Out of the four measures explored, the CEDV had been implemented in the most LMICs (although application was still sparse), met the most criteria for COSMIN PROMs, and most closely aligned with the definition of exposure to DVA used for the purpose of this paper, and so further work should be done on assessing its validity within LMICs, and potential to accept this as a standardized scale.

An accepted standardized measure for childhood exposure to DVA would undoubtedly allow for a more effective, and empirically sound comparison of the number of children exposed to DVA across a wider range of populations. This would provide a more accurate estimate of both the number of children who are exposed to DVA globally, along with who is more likely to be exposed so interventions can be targeted to and implemented with those most at-risk.

## Author contributions

The study was conceptualized by RH through discussions with SM and AC. RH conducted the literature search and critical appraisal and wrote the main draft of the article. SM and AC commented and reviewed all versions. All authors contributed to the article and approved the submitted version.
