# Supplementary material for: Childhood exposure to domestic violence: can global estimates on the scale of exposure be obtained using existing measures?
Source: Front Public Health. 2024 May 22;12:1181837. doi: 10.3389/fpubh.2024.1181837 (PMC11150823; doi:10.3389/fpubh.2024.1181837)
Supplement: Supplementary file 1 [file Table_1.DOCX]

Supplementary Material

Childhood exposure to domestic violence: can global estimates on the scale of exposure be obtained using existing measures?

Rebecca Harris^1*^, Andrew Amos Channon^2†^, Sara Morgan^3†^

^1^ Centre for Global Health and Policy, Department of Social Statistics and Demography, University of Southampton, Southampton, United Kingdom

^2^ Centre for Global Health and Policy, Department of Social Statistics and Demography, University of Southampton, Southampton, United Kingdom

^3^ Centre for Population Health Sciences, Faculty of Medicine, University of Southampton. Southampton, United Kingdom

^†These authors contributed equally to this work and share last authorship^

*** Correspondence:**Rebecca Harris
RJ.Harris@soton.ac.uk

# Appendix A. Summary of measures exploring childhood exposure to domestic violence and abuse

Note: Some of these measures also explore direct victimization, or violence in another domain (e.g., peer-to-peer violence, exposure to community violence). For the purpose of this table, only information related to exposure to DVA has been included. The language used in this table reflects that used within the respective measure.

| **Measure** | **Year of Publication** | **Country of Origin (COI)** | **Population group the measure was designed for** | **Victim-Perpetrator Relationship Assessed (DVA)** | **Violence Domain** | **Exposure Timeframe** | **Type of DVA Measured**  **(physical, sexual, psychological/emotional, coercive control, economic abuse, harassment and stalking, online/digital abuse)** | **Type of Exposure to DVA Measured**  **(Using Holden’s 2003 taxonomy)** | **Countries Implemented In**  **(Other than COI)** |
| --- | --- | --- | --- | --- | --- | --- | --- | --- | --- |
| Caregiver-report Modified version of the Severity of Violence Against Women Scales (34) | 2011  Adaptation of Marshall (1992; 35). | United States | Mothers with a child 1-3 years old | Mother-partner | Intimate Partner Violence | Lifetime | Physical, sexual, psychological | Victimized, eyewitness, overhears, observes the initial effects, experiences the aftermath, ostensibly unaware. | None found |
| Child Exposure to Domestic Violence Scale (36) | 2008 | United States | Children 10-16 years old | Female caregiver-partner | Exposure to domestic violence at home and in the community, the child’s exposure and involvement in the situation, presence of risk factors and exposure to other types of victimization  Specifically asks about school and community, on television or in a movie, video games, and other adults in their family that they do not live with. This includes questions on exposure and experiencing different types of violence and victimization. | Lifetime | Physical, psychological, coercive control | Eyewitness, overhears, observes the initial effects, hears about it, intervenes | Brazil (50)  Iran (52)  Iraqi-Kurdistan (53)  India (51)  Pakistan (54)  South Africa (55)  Spain (56)  Sweden (48,57,58) |
| Childhood Experiences of Violence Questionnaire (37) | 2008 | Canada | Children 12-18 years old | Caregiver-partner | Peer-to peer violence, exposure to domestic violence, physical violence and sexual violence | Lifetime; respondent provides information on the timeframe of exposure | Physical, sexual, psychological, emotional | Eyewitness, overhears | None found |
| Children's Perception of Interparental Conflict Scale (38) | 1992 | United States | Children 9-12 years old | Parent-parent  Exposure to interparental conflict and the relationship to child adjustment. | Exposure to IPV | Not specified | Physical, psychological/emotional | Eyewitness, overhears, intervenes | China (64-66)  Pakistan (67)  Portugal (68)  Spain (69) |
| Computer Assisted Child Maltreatment Inventory (39) | 2010 | United States | Adults over the age of 18 | Parent-parent | Exposure to IPV | Lifetime | Physical, verbal, psychological/emotional | Eyewitness, overhears, hears about it, observes the initial effects | None found |
| Family Aggression Screening Tool (40) | 2016 | England, United Kingdom | Children and young adults 16-24 years old | Parent (or adult)-opposite sex partner | Family violence and IPV exposure | Lifetime | Physical, emotional/psychological, verbal | Not specified, children are asked “if you remember this kind of aggression happening in your house when you were growing up” | None found |
| Juvenile Victimization Questionnaire (41) | 2005 | United States | Children 8-17 years old, caregiver of 2-9 year old child  The questionnaire is usable in interview format with children as young as age 8 and as old as age 17. It can be used in a self-administered format for juveniles 12 and older. There is also a “Caregiver version,” by which a caregiver could be interviewed as a proxy for a child, especially a child under age 8. Additionally, it can be adapted for retrospective reporting of childhood events by adult respondents. | Parent-opposite sex partner | Conventional crime, child maltreatment, peer and sibling victimization, sexual victimization, and witnessing/exposure to indirect victimization | Lifetime | Physical, sexual, psychological/emotional | Witnessing and indirect victimization | Australia (70)  China (71)  Israel (72)  Pakistan (73)  Portugal (74)  South Africa (75)  Spain (76)  UK (77) |
| Things I Have Seen and Heard (TISH) (42) | 1990 | United States | Children 6-14 years old | “Grownups in my home” | Exposure to violence at home and in the community | Lifetime | Physical and psychological | Witnesses and overhears | None found |
| Timeline Followback Interview – Children's Exposure to Partner Violence (43) | 2009 | United States | Couples with a child 6-16 years old (interviewed separately) | Caregiver-partner | Exposure to IPV | Past 90 days | Physical | Eyewitness, overhears, ostensibly unaware | None found |
| Violence Exposure Scale for Children (44) | 1995 | United States | Children aged 4-10 | “At home” | Exposure to violence at home, school, and/or the neighborhood | Not specified | Physical | Eyewitness | Israel (79)  The measure is also available in Spanish (80), although no studies using this measure could be found. |
